# Spinal Anaesthesia for Cesarean Section in a Patient with Vascular Type Ehlers-Danlos Syndrome

**DOI:** 10.1155/2018/1924725

**Published:** 2018-01-28

**Authors:** Jeffrey M. Carness, Mark J. Lenart

**Affiliations:** ^1^Department of Anesthesiology, United States Naval Hospital Yokosuka, Yokosuka, Japan; ^2^Department of Anesthesiology, Naval Medical Center Portsmouth, Portsmouth, VA, USA

## Abstract

We report the administration of spinal anaesthesia for cesarean delivery in a parturient with vascular Ehlers-Danlos syndrome. Parturients who genetically inherit this disorder are at risk for significant morbidity and mortality. Risks during pregnancy include premature labor, uterine prolapse, and uterine rupture. Additionally, such laboring parturients are at increased risk of hemodynamic volatility, vascular stress, and severe postpartum hemorrhage. Instrumented delivery and cesarean delivery bring additional risks. Nonpregnancy-related complications include excessive bleeding, intestinal rupture, cardiac valvular dysfunction, and arterial dissection. Despite the complexity of this condition, literature focusing on specific intraoperative anaesthetic management is sparse.

## 1. Introduction

Ehlers-Danlos syndromes (EDS) comprise connective tissue disorders affecting multiple organ systems to include the integumentary, musculoskeletal, pulmonary, digestive, and cardiovascular systems. These disorders are classified according to the Villefranche Nosology which was originally adopted in 1997 [1]. To date, six underlying types of disease have been identified and are categorised as follows: classical (formerly type I/II), hypermobility (formerly type III), vascular (formerly type IV), kyphoscoliosis (formerly type VI), arthrochalasia (formerly type VIIA/VIIB), and dermatosparaxis (formerly type VIIC) [1]. Each type possesses unique clinical manifestations and anaesthetic implications. Peripartum complications associated with vascular EDS include preterm premature rupture of membranes, arterial dissection/rupture (e.g., aorta, iliac, splenic, and coronary), uterine rupture, uterine incision dehiscence, 3rd-/4th-degree lacerations, and postpartum hemorrhage [2]. Peripartum mortality rates are highly variable and range from 4.3% to 25% [2]. Case reports in the literature specifically focusing on the intraoperative anaesthetic management of these patients are sparse [3, 4]. Here, we report the perioperative anaesthetic management of a parturient with vascular EDS. The patient provided written permission for publication of the report.

## 2. Case Description

The patient was a 23-year-old female (Gravida 1, Para 0) presenting for obstetric care at eight weeks gestational age with genetic documentation of a* COL3A1* mutation confirming vascular type EDS. She reported emergency room visits for significant hematomas in addition to current treatment with losartan for a history of cerebral aneurysm. First trimester brain MRI revealed full resolution of the aneurysm. Her second trimester transthoracic echocardiogram was unremarkable. A complete blood count, comprehensive electrolyte panel, and electrocardiogram were also unremarkable. Hemoglobin and hematocrit concentrations were 9.2 g·dl^−1^ and 28.9%. Prothrombin time, partial thromboplastin time, and platelet function analysis were within normal limits. We planned for surgical delivery at 34 + 0 weeks of gestational age. This gestational age represented a balance of fetal risk due to premature delivery and maternal risk due to increasing fetal size and an active second stage of labor. We elected for an ultrasound-guided arterial catheter, central venous catheter, and spinal anaesthetic.

A multidisciplinary team including physicians from the blood bank, cardiologists, cardiothoracic and vascular surgeons, neonatologists, and obstetric anaesthesiologists was assembled. On the day of surgery, the patient presented to the labor and delivery ward with normal vital signs and a reassuring airway. Two 18-gauge peripheral IV catheters were placed. The surgical procedure was performed in the cardiothoracic/vascular operating suite, where infusions of 1 g tranexamic acid and 0.3 *μ*g·kg^−1^ deamino-D-arginine vasopressin (DDAVP) were initiated upon patient arrival. While in the supine position, a 20-gauge catheter was introduced into the right radial artery under ultrasound guidance. The patient was then placed in a sitting position for placement of the spinal anaesthetic where 2+ pitting edema was noted. A curvilinear low-frequency ultrasound probe was obtained and the lumbar anatomy visualised. After sterile preparation and draping, a 25-gauge 3.5′′ Pencan® Spinal Needle was advanced at the L3/L4 interspace. Following subarachnoid puncture, 13.5 mg of 0.75% bupivacaine, 100 *μ*g of epinephrine, 30 *μ*g of clonidine, and 100 *μ*g of morphine were injected. She was then placed in the supine position and the femoral region prepared and draped aseptically. The femoral vein was cannulated with a triple lumen catheter under ultrasound guidance. Spinal level was verified at T6 prior to bladder catheterization and subsequent uncomplicated cesarean section. Blood loss was 700 ml and the patient's postoperative hemoglobin and hematocrit concentrations were 7.2 g·dl^−1^ and 22.9%. Twenty units oxytocin in 1-liter normal saline was given via IV infusion. Following the procedure, the patient was transferred to the intensive care unit to utilise the combined resources of obstetric and critical care nursing. Hourly neurologic checks were performed to monitor for symptoms of a neuraxial hematoma. Maternal postoperative pain management was dictated by the surgeon and included the use of a patient-controlled analgesic device. Maternal complications were limited to an ileus requiring nasogastric tube placement and a brief extension of her hospital stay. The ileus resolved without complication permitting discharge to home within one week of surgery. During phone follow-up two weeks later, the patient reported satisfaction with her anaesthetic and denied any complaints or concerns.

## 3. Discussion

Vascular EDS is a single subtype of a heterogeneous collection of connective tissue disorders associated with mutations in the genes that code for the production of collagen. Vascular EDS is associated with mutations in type III procollagen (*COL3A1*: OMIM #120180). The diagnosis, which carries a significant risk of peripartum morbidity, requires both genetic identification and the presence of at least two of the following major clinical criteria: “(1) arterial, gastrointestinal or uterine fragility or rupture, (2) thin, translucent skin without hyper-elasticity, (3) extensive bruising, or (4) characteristic facial appearance (thin lips and nose, hollow cheeks, large eyes, small chin)” [1]. Vascular EDS represents less than 10% of all EDS patients and carries a relatively low prevalence of 1 : 100,000–1 : 200,000 patients [5]. As such, few case reports have addressed the anaesthetic management of parturients with vascular EDS [3, 4]. Our preoperative deliberations focused on options for vascular access (peripheral versus central; standard peripheral IV versus rapid infusion catheter; multilumen central venous access versus large-bore introducer), blood pressure monitoring (noninvasive versus intra-arterial; radial versus femoral arterial), anaesthetic approach (general versus spinal versus epidural anaesthesia), neuraxial anaesthetic technique (single injection versus continuous administration of local anaesthetic), blood management (type of blood products; intraoperative cell salvage), and coagulation (antifibrinolysis).

In this context, we reviewed a publication which reported the management of a vascular EDS patient undergoing elective cesarean section at 36 weeks of gestational age [4]. Coagulation studies, complete blood count, and transthoracic echocardiogram were obtained preoperatively. Vascular access included two large-bore IV catheters, a radial arterial catheter, and an antecubital central venous catheter. Volume preloading consisted of 1-liter crystalloid and 0.5-liter colloid solutions. Tuohy needle-guided spinal anaesthesia was established by subarachnoid injection of 2.8 ml of 0.5% bupivacaine via a 25-gauge Whitacre spinal needle. An epidural catheter was inserted for postoperative analgesia.

Another report described successful continuous epidural anaesthesia with ropivacaine and fentanyl administered via a 20-gauge polyurethane catheter for labor and forceps delivery in a patient with vascular EDS [3]. In the absence of further case reports specific to the anaesthetic management of vascular type EDS parturients, we referenced an algorithm by Wiesmann et al. suggesting a preoperative approach to the EDS patient [6]. Specifically, we confirmed the genetic subtype, discussed prior EDS complications, and confirmed we had prolonged postoperative care facilities available. We focused extra care on patient positioning, cross-matched the patient for blood products, and administered desmopressin intraoperatively due to the patient's history of recurrent hematomas. We pursued a multidisciplinary approach and were influenced by these preoperative recommendations. We further obtained a first trimester brain MRI to evaluate for cerebral aneurysm and a second trimester transthoracic echocardiogram to evaluate for aortic root widening, aortic aneurysm, or valvular pathology. We noted several factors in considering the intraoperative approach to her cesarean delivery. Risks of general anaesthesia include the difficulties of tracheal intubation and the potential complications encountered in vascular EDS patients (i.e., arterial dissection/cerebral hemorrhage during hypertensive response to intubation, possible pneumothorax from positive pressure ventilation, and unstable cervical spine with potential for atlantoaxial subluxation) [3, 7, 8]. Alternatively, neuraxial anaesthesia is potentially less consistently reliable and may increase the risk of epidural hematoma [7]. Though the majority of vascular EDS patients have normal coagulation, there is a tendency toward prolonged bleeding and platelet dysfunction [9]. Furthermore, the vascular fragility combined with the tendency for platelet dysfunction, and in this patient the additional history of recurrent hematomas, raised our concern for an increased risk of epidural hematoma. After counseling, however, the patient chose a spinal anaesthetic, desired her spouse to be in the operating room at delivery, and requested to be awake at delivery. No cases of neuraxial hematoma in a parturient with vascular EDS were noted during literature review. Explanations for a negative literature review likely include the extremely low incidences of both vascular EDS and neuraxial hematoma. Due to the risk of platelet dysfunction, postpartum hemorrhage, difficult uterine closure, and vascular fragility and in the setting of relatively benign side effect profiles, we elected to administer tranexamic acid and DDAVP [10, 11]. Historically, both medications have been used to mitigate the potential for postpartum hemorrhage. We considered the risk and benefit of administering these medications with the risk and benefit of the patient receiving an autologous blood transfusion. Ultimately, we selected a single spinal injection and a small 25-gauge spinal needle to minimise tissue trauma. We added epinephrine and clonidine to the local anaesthetic solution to lengthen the duration of the nerve block. We used invasive arterial monitoring with central venous access to allow for the potential use of vasoactive agents and/or blood product administration, should the loss of peripheral IV access occur. Due to the patient's history of hematoma formation, we chose femoral vascular access. The literature does not support an optimal site for central venous access in this patient population. Though a peripherally inserted central catheter (PICC line) provides continuous IV access, it is unreliable for resuscitation. In the event of significant vascular trauma with bleeding, the subclavian is noncompressible and the internal jugular's juxtaposition to the airway is undesirable. The femoral site is, however, recognizably not visible to the anaesthetist during the procedure. Of note, urgent interventional radiology, if necessary, remains possible using the contralateral vasculature. Abiding by National Institute for Clinical Excellence (NICE) guidelines, and in line with recommendations from Wiesmann et al., we employed ultrasound-guided techniques for all of our invasive procedures in effort to reduce the risk of vascular injury and neuraxial hematoma.

In conclusion, we have presented the safe and uneventful delivery of a 34-week gestational aged parturient with documented vascular EDS who underwent primary elective cesarean section under spinal anaesthesia. In this rare case, which had significant anaesthetic implications, the potential for morbidity was great. However, our patient had none of the complications documented in the literature, suggesting that this patient may have done well with any number of anaesthetic alternatives.
